# Liquid Biopsy in Hepatocellular Carcinoma: ctDNA as a Potential Biomarker for Diagnosis and Prognosis

**DOI:** 10.1007/s11912-025-01681-3

**Published:** 2025-05-09

**Authors:** William Yang, Romario Nguyen, Fatema Safri, Muhammad J. A. Shiddiky, Majid E. Warkiani, Jacob George, Liang Qiao

**Affiliations:** 1 Storr Liver Centre, The Westmead Institute for Medical Research, The University of Sydney and Westmead Hospital, Westmead, NSW 2145 Australia; 2 Rural Health Research Institute (RHRI), Charles Sturt University, Orange, NSW 2800 Australia; 3 School of Biomedical Engineering, The University of Technology Sydney, Ultimo, NSW 2007 Australia; 4 Storr Liver Centre, Westmead Institute for Medical Research (WIMR), the University of Sydney, Westmead, NSW 2145 Australia

**Keywords:** Hepatocellular carcinoma (HCC), Circulating tumour DNA (ctDNA), Circulating tumour cells (CTCs), Cell-free DNA (cfDNA)

## Abstract

**Purpose of Review:**

Hepatocellular carcinoma (HCC) is a leading cause of cancer-related mortality worldwide, with rising incidence and mortality. Early-stage HCC is often asymptomatic, and the lack of reliable early diagnostic markers leads to late-stage diagnosis with limited treatment options. Current treatment relies on tumour staging and patient status, but accurate staging requires invasive procedures that fail to capture tumour heterogeneity and progression. There is an urgent need for less invasive diagnostic strategies, such as liquid biopsy technologies, which allow for repeated sampling and real-time analysis of tumour dynamics. Liquid biopsies, including circulating tumour cells (CTCs) and circulating tumour DNA (ctDNA), offer the potential to monitor recurrence, metastasis, and treatment responses, potentially transforming HCC clinical management by enabling earlier intervention and personalised treatment strategies.

**Recent Findings:**

Recent studies emphasise the potential of ctDNA as a non-invasive biomarker by targeting DNA methylation for early HCC detection, enabling timely intervention and personalised treatment to improve patient outcomes. Comparative analyses have shown that ctDNA mutation testing outperforms alpha-fetoprotein (AFP), with a sensitivity of 85% and a specificity of 92%, compared to 60% sensitivity and 80% specificity for AFP. Additionally, profiling the ctDNA mutation landscape of 100 HCC patients has identified recurrent mutations in genes such as TP53, CTNNB1, and AXIN1.

**Summary:**

ctDNA appears to be a promising non-invasive biomarker in the clinical management of HCC patients, with the sensitivity and specificity improving by 41.67% and 15% respectively. The ctDNA mutations, particularly those targeting DNA methylation, highlight great potential for precision medicine, critical for early diagnosis and prognosis of HCC.

## Introduction

Hepatocellular carcinoma (HCC) is a major contributor to cancer mortality and is the sixth most prevalent cancer globally as of 2023 [1]. In Australia, the incidence and mortality rates of HCC have been on the rise. According to the Australian Institute of Health and Welfare (AIHW) Cancer report, the age-standardised incidence rate for HCC increased from 10 cases per 100,000 patients in 2019 to 12 cases per 100,000 patients in 2023 [2]. Between 2021 and 2023, the age-standardised mortality rate went from 9.2 deaths per 100,000 patients to 9.7 deaths per 100,000 patients respectively [2]. Early diagnosis and timely intervention are the key to treatment success since early-stage HCC may allow curative therapies such as surgical resection, liver ablation or transplantation. When detected and treated at an early stage, the 5-year survival rate for HCC is reported to range between 50% and 70% [3]. However, only 5–10% of HCC patients in Western countries and approximately 30% in Asian countries are diagnosed at these early stages [4]. For patients with advanced HCC, therapeutic options remain severely limited [5]. These statistics underscore the urgent need for reliable biomarkers to enable the early detection of HCC.

Current diagnostic tools for HCC, including medical imaging, serum alpha-fetoprotein (AFP) levels, and tissue biopsies, have notable limitations [6]. Conventional biopsies are invasive, prone to sampling errors, and unsuitable for repeated use. Serum markers, while minimally invasive, lack the sensitivity to reliably detect early-stage HCC or monitor tumour recurrence [3]. In contrast, liquid biopsies present a transformative opportunity to address these limitations. As non-invasive tools, liquid biopsies enable repeated sampling, provide comprehensive genetic and molecular profiles of primary and metastatic tumours, and facilitate the real-time monitoring of tumour evolution [7]. As such, liquid biopsies are increasingly recognised as a superior alternative to traditional invasive tests and serum biomarkers in disease diagnosis and monitoring [8, 9].

Most often, liquid biopsies utilise non-solid tissue samples such as blood and other body fluids (e.g., cerebrospinal fluid, saliva, ascites, stool and urine samples) for disease diagnosis and monitoring [4]. Key elements of liquid biopsies typically include circulating tumour cells (CTCs), circulating tumour DNA (ctDNA), cell-free microRNAs and extracellular RNAs (e.g., tumour-educated platelets) and exosomes. These materials carry the molecular and genetic signatures of the tumour tissues and can thus yield critical insights. For instance, analysing therapeutic targets and mutations in drug resistance-conferring genes within CTCs and ctDNA enhances our understanding of disease mechanisms and facilitates the development of personalised treatment plans. In recent years, advances in liquid biopsy technologies have enabled more precise and individualised approaches to HCC management, particularly in early detection, prognosis, and treatment response prediction.

**Fig. 1:**
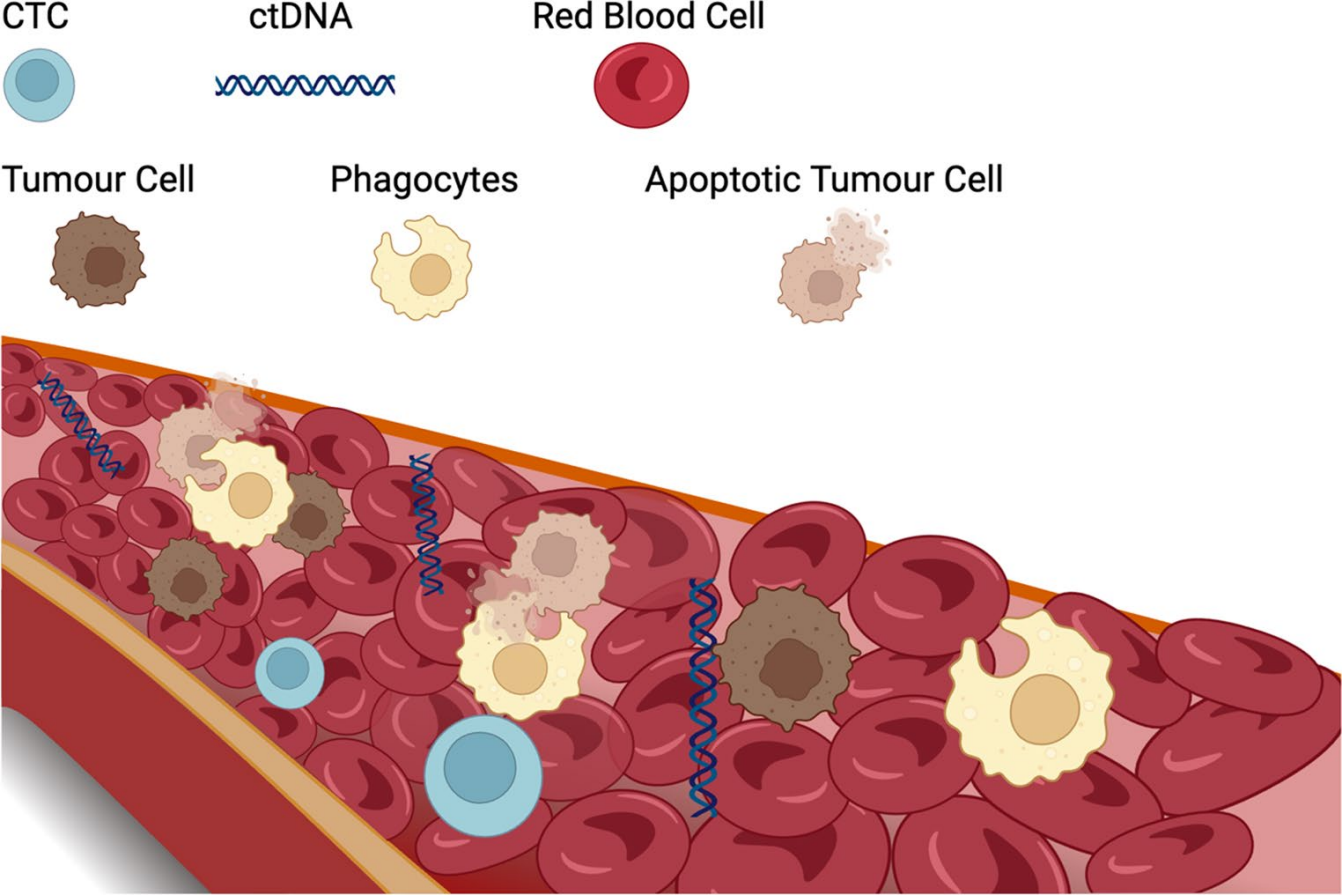
Peripheral blood containing CTCs and ctDNA. CTCs and ctDNA are the major elements in liquid biopsy. CTCs are cancerous cells shed from metastatic or original tumours and found circulating through the bloodstream, serving as “grains” that can potentially cause metastases. CtDNA comprises small DNA fragments shed by malignant cells into the peripheral blood. This DNA analyte could serve as a real-time molecular profile of the tumour. Created with Biorender.com

In this review, we will focus on the two principal components of liquid biopsies: CTCs and ctDNA (Fig. 1) by summarising their applications in the diagnosis, prognosis, and management of HCC, highlighting their potential to transform the clinical landscape of this devastating disease.

## Circulating Tumour Cells (CTCs)

CTCs are cancer cells shed from original or metastatic tumours that have spread and then circulate freely in bloodstream. CTCs are extremely rare, typically amounting to only several hundred cells per millilitre depending on the method of detection/isolation and definition used. CellSearch™ currently defines a CTC as “any nucleated circulating cell larger than 4 µm that expresses EpCAM, Cytokeratins 18, 19, or 20” [5]. While CTCs are difficult to capture from whole blood samples, they provide invaluable insights into the genetic information of the original tumours such as DNA, RNA and proteins profiles. The clinical utility of CTCs in early detection of cancers is limited due to their extremely low numbers and short lifespan (CTCs typically last 1 to 2 h in the blood) during the early stages of cancer [6]. Multifactorial factors may contribute to the rarity of the CTCs, including rapid apoptosis after shedding, cellular damage induced by hemodynamic shear force in blood stream, attacks from the immune system and in case of chemotherapy, targeting drugs-induced killing [10]. It was estimated that less than 0.01% of CTCs that enter circulation survive and result in metastasis, thus restricting their application as an early cancer detection tool [7]. Nevertheless, the presence of CTCs after primary tumour resection indicates tumour recurrence [8]. Highly sensitive detection techniques are needed to increase clinical utility of CTCs in the early diagnosis and disease monitoring of cancers.

### Circulating Tumour DNA (ctDNA)

Circulating tumour DNA (ctDNA) is a type of cell-free DNA (cfDNA) in the blood stream released from necrotic and apoptotic cells [9]. Autophagy of malignant cells, activated as a survival strategy to counter apoptosis, can release ctDNA [11]. ctDNA may also be released from hypoxic cells [12] as well as from the primary or metastatic cancers [13]. The most significant sources of ctDNA include necrotic or apoptotic tumour cells which release fragmented DNA into the blood [5, 14, 15]. In principle, the genetic information of ctDNAs remains identical to that of their original cell source. However, ctDNA only accounts for a very small portion of total cfDNA, and its presence usually poses little cancer risks.

Currently, classical testing cannot distinguish ctDNAs from other DNA types found in circulation. Only mutations specific to tumour cells on cfDNA can indicate its presence [16]. CfDNA, commonly found in serum or plasma, typically consists of double-stranded DNA molecules. Mutated molecules tend to be shorter than their non-mutant counterparts [16]. A study conducted by Mouliere et al., showed the cancer-specific ctDNA molecules typically consists of 167 base pairs (bp). However, fragments between 90 and 150 bp were found to have better detection rate [17]. It is interesting to note that cancer patients tend to possess higher concentrations of normal DNA than healthy individuals, which may dilute ctDNA when treatments such as chemotherapy or radiotherapy are administered [13]. To mitigate this in HCC, plasma or serum samples could be taken prior to each treatment and over multiple time points in capturing peak levels of ctDNA concentration. CfDNA has an extremely short half-life of between 16 min and 2.5 h [18]. Cancer-associated molecular features that may help differentiate the ctDNAs from normal circulating DNA include single nucleotides [19–21] and CpG methylation changes [22–24].

### Current Methodologies for Detecting CTCs and ctDNA in HCC Patients

CTCs and, more recently, ctDNA have been detected with increasing frequency in HCC patients. The characteristics and efficacy of these detection methodologies are summarised in Tables 1 and 2, respectively.

**Table 1:** CTC detection rates in blood samples from HCC patients

No. of cases	Methodology	Detection rate (%)	References
20	CellSearch™	40	Kelley et al. [25]
299	CellSearch™	42.6	Guo et al. [26]
42	CellSearch™	52.3	Fang et al. [27]
139	CellSearch™	54.0	Yu et al. [28]
57	CellSearch™	15.8	von Felden et al. [29]
81	RT-PCR (mK19, mCD44)	22.2	Choi et al. [30]
112	CanPatrol™	90.18	Qi et al. [31]
69	ImageStream	65.2	Ogle et al. [32]

**Table 2:** CtDNA detection methods and identified targets in HCC patients

No. of cases	Methodology	Target/s	References
14	Next-generation sequencing (NGS)	MYC, CDK6, RAF1, EGFR, FGFR1, CCNE1, PIK3CA, BRAF	Ikeda et al. [33]
	Single nucleotide mutation (SNV)	CDKN2A, PTEN, ARID1A, MET, CTNNB1, TP53	
48	Single nucleotide mutation (SNV)	TERT, CTNNB1, TP53	Huang et al. [34]
	Droplet digital PCR (ddPCR)		
41	Single nucleotide mutation (SNV)	TERT, CTNNB1, TP53	Liao et al. [35]
	MiSeq™ system		
40	Methylation specific PCR (MS-PCR)	HOXA9	Kuo et al. [36]
180	Methylation specific PCR (MS-PCR)	RASSF1A, COX2, APC	Lu et al. [37]
237	Pyrosequencing	TBX2	Wu et al. [38]
	Real-time PCR		
	Methylation specific PCR (MS-PCR)		
98	Methylation specific PCR (MS-PCR)	SEPT9	Oussalah et al. [39]
26	Next-generation sequencing (NGS)	ARID1A, CTNNB1, TP53	Ikeda et al. [40]

### Techniques for CTC Analysis

Over the past decade, multiple CTC separation techniques have been developed. These can generally be divided into two categories: physical methods and biochemical methods. Physical methods make use of the physical characteristics of CTCs, such as size (filter-based devices), density, electric charge, migration capacity and deformability [41]. Biochemical methods primarily employ antigen-antibody interactions by applying specific antibodies to tumour-specific biomarkers such as epithelial-cell adhesion molecule (EpCAM), prostate-specific antigen (PSA) and human epidermal growth factor receptor 2 (HER2) [42]. EpCAM is the most commonly used antigen for CTC purification, as it is expressed on all epithelial cells but is absent in blood [43]. CellSearch™ is the only FDA-approved EpCAM-based technology for counting and capturing CTCs. This method isolates CTCs from blood using anti-EpCAM magnetic beads, followed by manual counting. However, it may fail to detect highly metastatic tumour cells undergoing epithelial-mesenchymal transition (EMT) [44]. To address this limitation, alternative approaches have been developed. These strategies involve initial enrichment using less specific physical methods, followed by targeted isolation technologies such as reverse transcription polymerase chain reaction (RT-PCR), flow cytometry, and fluorescence in-situ hybridisation (FISH) [45]. In HCC, EpCAM expression varies, with 20 − 52.5% cases testing positive [46, 47]. Thus, microfluidic technologies and CTC chips have been designed to improve CTC detection and enhance the efficiency of isolating rare CTCs [48]. Microfluidics-based CTC isolation offers advantages by leveraging both biological and physical differences between CTCs while minimising non-specific binding on functionalised surfaces [49]. However, other existing CTC isolation techniques require extensive validation before clinical implementation.

### Techniques for ctDNA Analysis

Only a very small portion (usually, < 0.01%) of total cfDNA is present in peripheral blood, where both quantitative and qualitative changes may occur. Detectable features of ctDNA within DNA structures include single nucleotide variations, copy number changes and methylation [45]. Recent innovations have facilitated the development of many sensitive and specific methods of detection, such as digital PCR [50], cancer personalised profiling by deep sequencing (Capp-Seq) [51], emulsion PCR-based BEAMing (beads, emulsions, amplification, and magnetics) [18], tagged-amplicon deep sequencing (TAM-Seq) [52], safe-sequencing system (Safe-SeqS) [53] for single nucleotide changes in ctDNA, as well as whole genome sequencing (WGS) to detect copy number variations. CtDNA analysis can be broadly classified into two main categories: targeted and untargeted. Targeted ctDNA analysis seeks to detect mutations within predefined sets of genes (such as *KRAS*), while untargeted analysis provides genome-wide screening to detect new genomic abnormalities that may contribute to resistance against specific targeted therapies [54].

### Principles and Advantages of ctDNA Detection Methods

#### Methylation-specific PCR (MSP)

MSP is a methodology that utilises PCR amplification of target genes to assess the methylation state of CpG islands [55]. Primers used in this approach are designed specifically for either methylated or unmethylated DNA sequences following bisulfite treatment, making this effective means of investigating patterns across diverse genes as well as quantifying ctDNA sequences that have undergone this modification. When integrated with real-time PCR, it helps researchers to simultaneously examine ctDNA patterns across numerous genes while quantifying ctDNA sequences.

#### Digital PCR (dPCR)

dPCR is a sensitive technique developed by Vogelstein and Kinzler for detecting low-level mutations [56]. They first used this approach as an enhancement of conventional PCR by isolating single DNA molecules via serial dilutions before testing for any mutations that may exist in them. Under ideal conditions, PCR amplification is performed under optimal conditions. Fluorescently labelled probes hybridised with fluorescently tagged amplicons enables the detection of sequence-specific PCR products and direct quantification of both alleles present in a sample using digital PCR. One study successfully used digital PCR to screen for PIK3CA, ESR1 and ERBB2 mutations for ctDNA in breast cancer reaching high sensitivity and specificity levels [57]. Digital PCR can be utilised in various methods including droplet-based systems, microfluidic platforms, beads, emulsions, amplification and magnetics [55].

#### Next-Generation Sequencing (NGS)

NGS has emerged as an invaluable method of examining ctDNA, providing rapid screening for mutations across a broad range of genomic regions across multiple cancer types [58]. Furthermore, targeted deep sequencing approaches such as TAm-Seq, Safe-Seq, Ion AmpliSeq and cancer personalised profiling by deep sequencing, have been used to ascertain sequence information on selected genomic regions present in plasma DNA [59], allowing novel means of analysing ctDNA profiles or patterns in depth.

MSP, dPCR and NGS are currently the three most widely used methods for detecting DNA aberrations. More specifically, dPCR is the preferred technique for identifying specific aberrations due to its high sensitivity and feasibility in identifying tumour-associated mutations at levels as low as 0.01% of ctDNA [21]. However, dPCR is limited to analysing specific foci at a given time. In contrast, NGS offers a broader genomic view through deep sequencing techniques such as Capp-Seq, TAM-Seq and Safe-SeqS [60], offering valuable insights for personalised medicine and cancer gene characterisation.

### Predictive Value of CTCs in HCC Metastasis and Recurrence

Although the definition of CTC positivity remains controversial due to various researchers using “≥1 CTC” or “≥2 CTCs” or “≥5 CTCs”, CTCs have long been recognised as accurate predictors of cancer metastasis and recurrence. However, detecting CTCs for HCC diagnosis remains challenging due to their low abundance, necessitating further research to establish their reliability in clinical settings [61]. Nevertheless, certain subsets of CTCs, such as EpCAM^+^ CTCs, have been recognised as predictive biomarkers for HCC prognosis. The presence of EpCAM^+^ CTC in HCC patients is associated with significantly shorter overall survival (OS) and a disease-free survival [28]. Studies have also shown a correlation between higher CTC counts and poorer patient prognosis [62]. Additionally, EpCAM^+^ CTC can serve as real-time indicator for tracking HCC recurrence. A preoperative CTC count of ≥ 2 per 7.5 ml has been shown to predict tumour recurrence post-surgery, particularly in patients with a low risk of recurrence, such as those with serum AFP levels at or below 40 ng/mL [63]. However, only 20 − 52.5% of HCCs cases express EpCAM^+^, limiting the applicability of EpCAM + CTCs as prognostic indicators and highlighting the need for further validation [46, 47].

To address the shortcomings of EpCAM^+^ CTCs in predicting HCC recurrence, researchers have explored the detection of CTCs expressing multiple markers. For example, the presence of EpCAM^+^ CTCs alongside CD4^+^ CTCs, CD25^+^ CTCs, Foxp3^+^ CTCs or Treg cells has been associated with HCC recurrence [64]. Patients with higher CTC/Treg concentrations have a significantly greater risk of postoperative recurrence compared to those with lower concentrations [64]. Other CTC subtypes have also been investigated as the predictors of early HCC recurrence. Wang et al. reported that mesenchymal and mixed CTCs were significantly more prevalent among HCC patients who experienced early recurrence than among those who did not. Furthermore, mesenchymal CTC positivity was identified as an independent risk factor for early recurrence [65]. In another study, HCC patients with CTC counts ≥ 16 and a mesenchymal CTC percentage of ≥ 2% were found to have an increased risk of lung metastasis, multi-intrahepatic cancer, and early recurrence [31].

### Predictive Value of ctDNA in HCC Survival

CtDNA serves as a valuable biomarker for predicting HCC survival, reflecting both early tumour detection and progression markers like metastasis. Increased levels of untargeted HCC ctDNA correlate with poor prognosis. Targeted ctDNA may better reflect intra-tumoural heterogeneity and more reliable predict poor prognosis more reliably [66]. The most frequently detected abnormalities in targeted ctDNA include hot spot mutations such as TP53, TERT and CTNNB1 [67]. Using dPCR, it was demonstrated that 56.3% of 48 HCC cases contained at least one type of these mutations, with mutant frequencies ranging between 0.33 and 23.7% [34], indicating significant intra-tumoural heterogeneity within HCC tumours. Using MiSeq technology, it was found that ctDNA mutations were more frequently detected among those with vascular invasion and predicted shorter disease free survival (DFS) (*p* < 0.001) [35]. Therefore, detecting HCC-associated mutations in ctDNAs can help overcome heterogeneity limitations and accurately predict HCC prognosis. However, circulating mutants may not come exclusively from tumour cells, as germline mutations can also be detected within plasma. Screening tumour samples prior to ctDNA analysis may facilitate accurate assessment of the source of mutation.

## Diagnostic Value of ctDNA in HCC

Lack of reliable screening strategies and early diagnostic tools for HCC remains an unmet clinical need. Current imaging modalities such as ultrasound, computed tomography (CT) and magnetic resonance imaging (MRI) allow clinicians to visualise HCC tumours, yet are unable to effectively differentiate benign from malignant lesions and provide molecular insights. However, ctDNA provides this advantage, allowing real-time monitoring of molecular profiles offering further advantages that may enhance HCC diagnostic accuracy. Hypermethylation of promoter regions has long been recognised as a critical contributor to carcinogenesis, as normal tissue DNA lacks or contains only minimal combinations of methylated tumour suppressor genes. Previous studies have shown that DNA methylation appears early during tumourigenesis and is reversible [7], providing an alternative way of early cancer detection. Since the methylation pattern of each cell type remains highly stable under physiological or pathologic circumstances [68], recognising different patterns of CpG methylation could provide discriminatory tools for HCC diagnosis. This means that DNAs specific to HCC can be highly specific and sensitive diagnostic markers for HCC.

In this aspect, Wong et al. [69] demonstrated that p15 and p16 methylation was present in the bloodstream of 92% of HCC patients. Subsequent studies in methylation profiles in HCC patients showed that promoter hypermethylation of the Ras association domain family 1 A (RASSF1A) was present in 90% of HCC cases, and this methylation pattern could help distinguish HCC from healthy controls or patients with chronic HCV infections, with a prediction accuracy of 77.5% and 72.5% respectively [70]. More studies have shown that abnormal promoter methylation within ctDNA may help detect early stage HCCs among high-risk populations, and combination of multiple methylated genes could increase the diagnostic power for HCC. For example, presence of the promoter methylation of RASSF1A, p15 and p16 in serum could detect HCCs with 94% specificity and 84% sensitivity [71]. Using another panel consisting of SFRP1, GSTP1 and RASSF1A genes, it was shown that presence of the methylated promotor region could accurately differentiate HCC cases from controls with 81.9% specificity and 92.7% sensitivity [72].

Alpha-fetoprotein (AFP) is currently the most widely used serum marker for HCC, but it has limited diagnostic value due to poor sensitivity (only 50% of HCC cases can be identified using this marker) [62]. Promotor methylation of HCC related genes in ctDNAs may help mitigate the poor diagnostic power of AFP. For example, detection of a panel of three abnormally methylated genes (RASSF1A, COX2, APC) in ctDNA could help detect nearly 75% of patients without AFP [37]. In another study, the presence of methylations within ctDNA could help distinguish HCC patients from those with other liver disease or healthy controls, and thus more reliably diagnose HCC than AFP [73]. Combination of the methylation status of β-actin with AFP in cfDNA could enhance HCC detection rate with a specificity of 94.4% and sensitivity of 95.1% [74]. It was also reported that the combined detection of HCC specific methylations in ctDNA with AFP significantly improves the diagnostic sensitivity of each individual marker [75, 76].

Other emerging biomarkers including Glypican-3 (GPC3), Des-gamma-carboxy prothrombin (DCP), and Osteopontin (OPN) also show promise in sensitivity and specificity [77]. GPC3 has demonstrated sensitivity and specificity rates comparable to AFP for early HCC detection, with studies reporting improved diagnostic accuracy when combined with AFP [78]. Similarly, DCP offered a sensitivity between 48 and 62% and specificity of 81–98% for early HCC diagnosis [79]. In OPN, the results were promising but not definitive due to the inconsistent cut-off values used [80]. As a result, further validation would need to be conducted before routine clinical use is adhered. The quantitative analysis of DNA methylation in ctDNA combined with other markers such as AFP, GPC3, DCP and OPN may improve the diagnostic power for HCC. However, a practical challenge is that an elevated ctDNA is difficult to define and as such, there is no standardised cut-off value for ctDNA as the detection methods for them vary amongst researchers.

### CtDNA for Tumour Surveillance and Early Diagnosis

Since liver biopsy is an invasive procedure, and for the diagnosis of advanced HCC it is unnecessary, ctDNA could serve as an efficient biomarker to track tumour progression dynamically and assess treatment efficacy, including the identification of mutations that cause acquired drug resistance as well as capture heterogeneity amongst tumours. Meanwhile, epigenetic changes caused by DNA methylation are linked to cancer development, and methylation patterns of ctDNA can facilitate the early diagnosis of HCC [81]. It has been confirmed that changes in the methylation patterns of several genes found in serum/plasma samples, such as INK4A, GSTP1, RASSF1A, p15 and p16, can help diagnose HCC. The level of DNA methylation in HCC tumours correlates closely with that found in its respective plasma ctDNAs [82], indicating the fidelity of ctDNA as a potential alternative for studying the DNA methylation pattern. In this aspect, Kotoh et al. [83] have produced a methylated SEPT9 test with 90% specificity and 63.2% sensitivity to detect HCC. Yan et al. [84] developed a panel of three tests combining cfDNA, age and AFP for the diagnosis of HCC, and demonstrated its superior accuracy compared to either of these tests alone.

ctDNA methylation not only can aid in diagnosis but can also serve as a predictor of disease prognosis. In this aspect, Kotoh et al. demonstrated that the copy number of methylated SEPT9 was related to BCLC stages, tumour size, tumour number and macrovascular invasions [83]. In another study, Kisiel et al. demonstrated that the elevated level of six methylated DNA markers (AK055957, ECE1, EMX1, CLEC11A, HOXA1 and PFKP) in ctDNA was closely linked to poor prognosis of HCC patients [85]. In a prospective cohort of patients with gastrointestinal tumours, cfDNA proved more reliable in detecting drug resistance gene mutations than tissue biopsies [86]. Additionally, ctDNA may provide valuable epigenetic profiles that provide surveillance and early diagnosis for treating HCC. Furthermore, testing the mutation status in ctDNA may provide valuable guidance for therapy selection. This guidance has led to the identification that PI3K/mTOR gene mutation in ctDNA was associated with decreased progression-free survival (PFS) in HCC patients treated with tyrosine kinase inhibitors, but not in the patients receiving immunosuppression treatments [87]. Thus, monitoring drug resistance genes in cancer patients could provide important guidance in therapy selection.

### Challenges and Perspectives

Liquid biopsy is an emerging non-invasive technique with transformative potential in early cancer diagnosis, monitoring disease progression, assessing treatment response, predicting recurrence, and determining prognosis. As much of the current evidence on liquid biopsy in HCC, the predominance of short-term studies outweighs longitudinal data. Recent technological advancements may provide continued long-term investigations on ctDNA, which is crucial to grasp its significance in cancer. The US National Laboratory of Medicine is currently conducting a clinical trial (NCT02838836) to evaluate the efficacy of CTCs and ctDNA in monitoring disease progression and treatment response in patients with solid cancers, including HCC. Among the many advantages of liquid biopsy, the analysis of epigenetic biomarkers in ctDNA holds particular promise for tracing the tissue of origin, thereby providing critical insights for cancer diagnosis. DNA methylation is the most frequently utilised epigenetic modification for altering ctDNA, as it displays highly tissue-specific effects that can be used to trace the original source of the tumour [88, 89]. Furthermore, deep sequencing of ctDNA can reveal nucleosome positioning and fragment-end patterns, providing additional clues about the tissue of origin [90].

Beyond cancer diagnosis, liquid biopsy particularly ctDNA analysis, offers great potential for classifying molecular subtypes of malignancies. For instance, plasma ctDNA has been used to classify transcriptionally defined subtypes of diffuse large B-cell lymphoma (DLBCL). A recent study found that ctDNA genotyping in 41 DLBCL cases was 88% consistent with tumour tissue biopsy classification, demonstrating its utility in guiding personalised therapeutic decisions [90]. Similarly, Martinez-Ricarte et al. developed a platform capable of simultaneously genotyping of six key genes (TERT, ATRX, IDH1, IDH2, HIST1H3B, H3F3A) in cerebrospinal fluid ctDNA, enabling subclassification and differentiation of diffuse gliomas - one of the most common brain tumours with heterogeneous prognosis and classifications [91]. The broad utility of ctDNA could classify HCC subtypes such as MYC vs. CDK6 mutants as illustrated by Ikeda et al. in Table II, guiding personalised therapies for HCC patients.

However, despite its promise, liquid biopsy faces several limitations. Cost remains a significant barrier, particularly for ctDNA-based diagnostics, which require advanced sequencing technologies, bioinformatics tools, and specialised expertise for interpretation. For example, conventional sequencing methods often demand high quantities of tumour-derived DNA to reliably detect mutations, limiting their application to cases with substantial tumour burden. Although PCR-based digital methods offer enhanced sensitivity for detecting somatic mutations in ctDNA, these techniques are expensive and require significant technical training. Furthermore, current assays are constrained by a sensitivity threshold, below which mutations may go undetected, resulting in false negatives.

Standardisation is another critical challenge for liquid biopsy. Variability in methodologies for isolating, enriching, and analysing CTCs and ctDNA from blood samples hampers reproducibility and clinical implementation. Current efforts by prominent organisations such as the national cancer institute (NCI) and european liquid biopsy society (ELBS) aims to develop standardised protocols and reference ctDNA tests across various patient cohorts, emphasising their need for validation. Multi-marker analyses may also provide a more comprehensive understanding of tumour heterogeneity and enhance the diagnostic utility of ctDNA [83]. To fully integrate liquid biopsy into routine clinical practice, large-scale, multicentre studies, and long-term clinical trials are essential. Most of the data supporting liquid biopsy have been generated from proof-of-concept studies, which, while promising, require further validation in real-world settings.

Despite these challenges, liquid biopsy is poised to become a cornerstone of precision medicine. Its non-invasive nature, ability to capture tumour dynamics in real time, and potential for guiding tailored therapies underscore its transformative potential. As technological advancements continue and clinical evidence accumulates, the integration of liquid biopsy into standard care is likely to redefine the diagnostic and therapeutic landscape for cancer patients.

### Future Perspectives

The future of ctDNA will continue to see growth in clinical practice in the coming years, specifically its detection through methylation analysis. Although the cost of dPCR, NGS technology and other sensitive detection techniques remains a significant factor, ctDNA deep sequencing will still become more widely employed for basic and clinical research studies. The integration of ctDNA methylation detection into other liquid biopsy technologies such as CTC analysis or extracellular versicles (EVs) analyses could increase diagnostic precision and clinical utility. Identification and refinement of existing marker panels will also play a vital role in further developing ctDNA methylation-based diagnostics. Multiomics data will increasingly become accessible, making comprehensive studies at multiple centres essential for understanding the clinical implications of patterns in ctDNA methylation. The potential of combining molecular biomarkers, traditional serum markers and imaging tools may provide the most cost-effective screening solution for HCC. By integrating ctDNA methylation, this breakthrough could enhance patient outcomes while decreasing healthcare costs and providing early detection, personalised treatments and effective disease surveillance.

## Conclusion

Genetic biomarkers that are frequently present in HCC could improve early diagnosis scores. With liquid biopsy, epigenetic and genetic biomarkers such as CTC/ctDNA provide another novel way of screening and tracking HCC. This approach holds great potentials, as it targets molecular features which are critical for early diagnosis of HCC. CtDNA fragmentation patterns for liver cancer diagnosis fit this profile as an ideal screening tool. We expect to witness exciting advancements in early HCC detection using liquid biopsy-based markers in coming years.

## Key References

- Pan A, Truong TN, Su YH, Dao DY. Circulating Biomarkers for the Early Diagnosis and Management of Hepatocellular Carcinoma with Potential Application in Resource-Limited Settings. Diagnostics (Basel). 2023; 13:676. DOI: 10.3390/diagnostics13040676.
-
  - ○ To date, this study explores various biomarkers, including alpha-fetoprotein, circulating tumour DNA (ctDNA), microRNAs, and proteins, evaluating their diagnostic performance and suitability for use in low-resource environments. They also highlighted the sensitivity and specificity of ctDNA and AFP, measuring a sensitivity of 85% and a specificity of 92%, while AFP exhibits a sensitivity of 60% and a specificity of 80%.
- Xu Y, Cai J, Zhong K, Wen Y, Cai L, He G, Liao H, Zhang C, Fu S, Chen T, Cai J, Zhong X, Chen C, et al. Plasma-only circulating tumor DNA analysis detects minimal residual disease and predicts early relapse in hepatocellular carcinoma patients undergoing curative resection. Front Oncol. 2023; 13:1119744. DOI: 10.3389/fonc.2023.1119744.
-
  - ○ This large study investigated the use of plasma-only circulating tumour DNA (ctDNA) analysis to detect minimal residual disease. They found that post-operative ctDNA detection was significantly associated with both MRD and recurrence, suggesting its potential as a non-invasive tool for monitoring HCC patients.
- Yang JC, Hu JJ, Li YX, Luo W, Liu JZ, Ye DW. Clinical applications of liquid biopsy in hepatocellular carcinoma. Frontiers Oncol. 2022; 12:781820. DOI: 10.3389/fonc.2022.781820.
-
  - ○ This study summaries the clinical applications of liquid biopsy in HCC, highlighting its potential for early detection, prognostic evaluation, and treatment guidance. The authors also suggest ctDNA mutations in TP53, CTNNB1, and AXIN1 can be detected using techniques like droplet digital PCR, MiSeq sequencing, and targeted sequencing.
- Bardol T, Pageaux GP, Assenat E, Alix-Panabières C. Circulating tumor DNA clinical applications in hepatocellular carcinoma: current trends and future perspectives. Clin Chem. 2024; 70(1):33–48. DOI: 10.1093/clinchem/hvad168.
-
  - ○ This new study highlights ctDNA as a potential candidate for early diagnosis, recurrence risk stratification, and prognosis evaluation, particularly when combined with traditional markers like alpha-fetoprotein.
- Yang J, Lin N, Niu M, Yin B. Circulating tumor DNA mutation analysis: Advances in its application for early diagnosis of hepatocellular carcinoma and therapeutic efficacy monitoring. Aging (Albany NY). 2024; 16(14):11,460. DOI: 10.18632/aging.205980.
-
  - ○ This study explores various ctDNA analysis methods, including PCR, next-generation sequencing, and droplet digital PCR (ddPCR), highlighting their potential for improved patient outcomes.
- Nakamura Y, Ozaki H, Ueno M, Komatsu Y, Yuki S, Esaki T, Taniguchi H, Sunakawa Y, Yamaguchi K, Kato K, Denda T. Targeted therapy guided by circulating tumor DNA analysis in advanced gastrointestinal tumors. Nat Med. 2024; (1):165–175. DOI 10.1038/s41591-024-03244-8.
-
  - ○ This large study of over 4,000 advanced cancer patients found that using ctDNA comprehensive genomic profiling can inform treatment decisions and improve patient survival.
- Zhu Q, Xie J, Mei W, Zeng C. Methylated circulating tumor DNA in hepatocellular carcinoma: a comprehensive analysis of biomarker potential and clinical implications. Cancer Treat Rev. 2024; 102,763. DOI: 10.1016/j.ctrv.2024.102763.
-
  - ○ This study discusses the clinical implications of ctDNA methylation for HCC screening, diagnosis, prognosis, and treatment response prediction.
- Manea I, Iacob R, Iacob S, Cerban R, Dima S, Oniscu G, Popescu I, Gheorghe L. Liquid biopsy for early detection of hepatocellular carcinoma. Front Med (Lausanne). 2023; 10:1218705. DOI: 10.3389/fmed.2023.1218705.
-
  - ○ This study emphasises the importance of early diagnosis due to limited treatment options for advanced HCC and suggests that combining liquid biopsy with traditional serum markers could improve diagnostic accuracy.
- Kopystecka A, Patryn R, Leśniewska M, Budzyńska J, Kozioł I. The Use of ctDNA in the diagnosis and monitoring of hepatocellular carcinoma—literature review. Int J Mol Sci. 2023; 24(11):9342. DOI: 10.3390/ijms24119342.
-
  - ○ This study discusses the potential of ctDNA analysis for early detection, personalised therapy selection, and treatment response monitoring, highlighting its advantages over traditional markers like alpha-fetoprotein.
- Mohamed YI, Lee SS, Demir T, Chamseddine S, Hu ZI, Xiao L, Elsayes K, Morris JS, Wolff RA, Hiatia R, Qayyum A. Circulating tumor DNA (ctdna) as a biomarker of response to therapy in advanced hepatocellular carcinoma treated with nivolumab. Cancer Biomark. 2024; 41(1):83–91. DOI: 10.3233/CBM-230431.
-
  - ○ This study investigated the prognostic significance of ctDNA in patients with HCC and treated with nivolumab, revealing that specific mutations PIK3CA and BRCA1 are associated with shorter overall survival (OS) and progression-free survival (PFS).

## Data Availability

No datasets were generated or analysed during the current study.
